# BMC Palliative Care reviewer acknowledgement, 2013

**DOI:** 10.1186/1472-684X-13-2

**Published:** 2014-02-04

**Authors:** Catia Cornacchia

**Affiliations:** 1 BioMed Central, Floor 6, 236 Gray's Inn Road, London, WC1X8HB, UK

## Abstract

**Contributing reviewers:**

The editors of BMC Palliative Care would like to thank all our reviewers who have contributed to the journal in Volume 12 (2013).

## 

Adriaan Visser

Nepal

Agnieszka Ardelt

USA

Anette Henriksson

Sweden

Anna Spathis

UK

Antonio Noguera

Spain

Arif Kamal

USA

Bernd Oliver Maier

Germany

Beverley Mcnamara

Australia

Brandi Vanderspank-Wright

Canada

Bridget Johnston

UK

Bruce Rumbold

Australia

Caprice Knapp

USA

Ceilito Reyes-Gibby

USA

Chris Feudtner

USA

Christian Schulz

Germany

Christoph Ostgathe

Germany

Christopher J. Mackinnon

Canada

Clare Gardiner

UK

Claudia Bausewein

Germany

Colette Reid

UK

Colin Campbell

UK

Daniela Cristina Stefan

South Africa

Dave Lu

USA

David Vickers

UK

David Hui

USA

David Oliver

UK

Deborah Carr

USA

Deborah Parker

Australia

Diana Jackson

UK

Dirk Houttekier

Belgium

Eleanor Wilson

UK

Finella Craig

UK

Gunn Grande

UK

Hamish Ross

UK

Jeff Myers

Canada

Joachim Cohen

Belgium

Jonathan Waite

UK

Joris Gielen

Belgium

Jos Schols

Netherlands

Joseph Amon

USA

Judith Paice

USA

Julia Downing

Uganda

Karen Brombley

UK

Karen Cox

UK

Karen Forbes

UK

Kate Flemming

UK

Katherine Froggatt

UK

Kathleen Lyons

USA

Keith Swetz

USA

Kristin Kieselbach

Germany

Larry Librach

Canada

Laura Funk

Canada

Laura Goodwin

UK

Lauren Rayner

UK

Lucy Selman

UK

Lud Van Der Velden

Netherlands

Lydia Bird

UK

Marc Roelands

Belgium

Maria Friedrichsen

Sweden

Mark Boughey

Australia

Martin Weber

Germany

Matthew Loscalzo

USA

Melissa Bloomer

Australia

Michael Harlos

Canada

Mitesh Rao

USA

Mitsunori Miyashita

Japan

Monika Führer

Germany

Nancy Preston

UK

Natalie Bradford

Australia

Natasja Raijmakers

Netherlands

Pat Sartori

UK

Paul Sinfield

UK

Paul Howard

UK

Paul Hutchison

USA

Paul Paes

UK

Paula Smith

UK

Peter Saul

Australia

Peter Fenwick

UK

Priska Bützberger Zimmerli

Switzerland

Renea Beckstrand

USA

Rhian Gabe

UK

Richard Harding

UK

Richella Ryan

UK

Robin Cohen

Canada

Ruth Davies

UK

Sarah Galbraith

UK

Sarah Hoare

UK

Saran Yoshida

Japan

Sheila Payne

UK

Silke Steinbach

Germany

Sriram Yennu

USA

Stefan Wirz

Germany

Steffen Eychmueller

Switzerland

Stephen Barclay

UK

Stuart Milligan

UK

Susan Mcclement

Canada

Susanne Jaquelin Johannes Claessen

Netherlands

Tabitha Thomas

UK

Takuya Shinjo

Japan

Tara Albrecht

USA

Tony Ryan

UK

Tony Obrien

Australia

Una Macleod

UK

Victoria Simms

UK

Wadih Rhondali

France

Yoshiyuki Kizawa

Japan

Yvonne Engels

Netherlands

## Pre-publication history

The pre-publication history for this paper can be accessed here:

http://www.biomedcentral.com/1472-684X/13/2/prepub

